# International dissemination of Brazil’s public health system and its technical cooperation initiatives for sickle cell disease, 2006-2010

**DOI:** 10.1590/S0104-59702025000100039en

**Published:** 2025-09-22

**Authors:** Juliana Manzoni Cavalcanti

**Affiliations:** i Researcher, Casa de Oswaldo Cruz / Fiocruz . Rio de Janeiro – RJ – Brazil juliana.manzoni@fiocruz.br

**Keywords:** Brazil, Sickle cell disease, South-South cooperation, Global health, Unified Health System (SUS).

## Abstract

This study is aligned with other research that critically analyzes the way sickle cell disease has been framed as a global burden to health since the 2000s. To this end, the study investigates Brazilian attempts to establish structural technical cooperation programs in health with Benin, Ghana, and Senegal, based on the comprehensive care model offered to people with sickle cell disease under its public health system, the Unified Health System (Sistema Único de Saúde, SUS). This international initiative is part of a broader drive to disseminate internationally the values of universalism and equality, enshrined in the Brazilian health reform and embodied in SUS.

## ANALYSIS

In late 2006, the physician Joice Aragão de Jesus gave a presentation at the third Congress of the International Organization for the Fight against Sickle Cell Disease about the comprehensive healthcare model devised for people with sickle cell disease within the scope of the Brazilian public health system, Unified Health System (Sistema Único de Saúde, SUS). The event, organized by the first ladies of the African Union, aimed to reach a consensus on a priority global policy for the disease – a policy focused on birth control that had already begun to take shape in different documents published by the World Health Organization (WHO) and the United Nations Educational, Scientific and Cultural Organization (Unesco, 2005; WHO, 2006, 2010a).

At the time, Joice Aragão de Jesus had been in charge of the National Policy for Comprehensive Care for People with Sickle Cell Disease and Other Hemoglobinopathies (Política Nacional de Atenção Integral às Pessoas com Doença Falciforme e Outras Hemoglobinopatias, PNAIPDF) in Brazil for about two years. In her presentation, she sparked a controversy when she explained that Brazil invested in comprehensive care for people with sickle cell disease and not in a family planning policy. Her words caused the suspension of the signing of a memorandum of understanding that would have supported reproductive control for people with sickle cell trait and disease (Jesus, Cortez, 14 maio 2024). After this event, Brazil sent representatives involved in the national policy covering the disease, PNAIPDF, and delegates from the Ministry of Health and the Brazilian Cooperation Agency of the Ministry of Foreign Affairs to Jamaica and to countries in sub-Saharan Africa to present the model of comprehensive care offered to people with sickle cell disease as part of SUS (Brasil, 2012).

The aim of this article is to contribute to the growing number of critical analyses of the way sickle cell disease has been framed as a global burden to health in the twenty-first century. To this end, I have investigated some of Brazil’s technical cooperation initiatives for comprehensive health for people with sickle cell disease, based on the SUS model, with sub-Saharan countries where the prevalence of the disease is high. Comprehensive care means engaging in actions to prevent disease, promote health, and provide support and recovery, focusing on the needs of the whole person. In this approach, the individual should be understood in their historical, political, social, and family context and receive care throughout their life, beginning with primary health care (PHC) and extending to more complex care and services (secondary and tertiary care) (Hartz, Contandriopoulos, 2004).

More broadly, in its foreign policy Brazil was giving South-South cooperation a new lease of life with a view to strengthening the country’s position in a multipolar world (Almeida, 2017). The cooperation initiatives analyzed in this article, between Brazil and Benin, Ghana, and Senegal, running from 2006 to 2010, can also be seen as endeavors to spread to other countries the values of universalism and equality enshrined in the Brazilian health reform, forming the institutional pillars of SUS.

During the same period, a global assemblage was taking shape with the support of scientists, managers, patient associations, and the WHO, which defined sickle cell disease and other hemoglobinopathies as preventable birth defects (Chattoo, 2018). Arguments for the social and reproductive control of hemoglobinopathies have primarily been supported by genetics. When geneticists take as paradigmatic the cases of thalassemia in Cyprus and Sardinia, which have small populations and have had prevention policies for decades, they project these contexts onto much broader, more complex realities, such as those encountered in India (Chattopadhyay, 2006). It is common knowledge that this reductionist interpretation is gaining prominence in the debate on global health to the detriment of a broader understanding, based on the importance of PHC and comprehensive monitoring to care for people with hemoglobinopathies.

Analyzing this new trend in genetics in relation to hemoglobinopathies requires investigating specific contexts in which the discourse of reproductive control has received a positive or negative reception. Following Burawoy et al.’s (2000) notion of the grounding of globalization and Biehl and Petryna’s (2013) call to ensure that people are at the center of studies on global policies, I analyze the role of certain actors in Brazilian foreign policy on health and the new way sickle cell disease is being framed medically and socially in the early twenty-first century to understand Brazil’s cooperation initiatives with Benin, Ghana, and Senegal between 2006 and 2010. Through oral history ^1^ and document analysis, I show how the Brazilian model of comprehensive care offered by SUS to people with sickle cell disease could undermine efforts to build a consensus around the adoption of restrictive health policies for people with sickle cell trait and disease and, at the same time, contribute to the dissemination of the values of Brazilian health reform, such as universality and equity.

The article is divided into three sections. In the first, I draw on the scientific literature, especially the work of Chattoo (2018), and analyses of WHO documents and scientific reports, to provide a brief overview of the emergence of this new discourse surrounding sickle cell disease as a threat to global health. In the second section, I address the international dissemination and promotion of the principles of equity and universality enshrined in the Brazilian health reform and embodied in SUS, analyzing the literature and the short-lived Health Reform Dissemination and Exchange Program (Programa de Difusão e Intercâmbio sobre a Reforma Sanitária, PRODIRS). In the third section, I complete my argument by looking at the technical health cooperation initiatives carried out by Brazil with Benin, Ghana, and Senegal from 2006 to 2010. I situate these initiatives in the broader history of Brazilian structural cooperation in health in the early 2000s, supported by the country’s South-South cooperation policy, and demonstrate how Brazil’s actions were important for fostering discussion about or implementing a new health strategy in the countries in question, shifting the focus away from sickle cell disease itself and towards the people affected by it and thereby contributing to a new appreciation of comprehensive care as a healthcare strategy.

### Global policies for sickle cell disease, genetics, and the World Health Organization

Since the beginning of the twenty-first century, sickle cell disease has been considered a global burden and threat. It is argued that increased migration to Europe has brought many individuals with sickle cell disease and trait, which therefore places a heavy burden on European health systems, while also, according to Weatherall and Clegg (2001) and Weatherall (2007), spreading the gene among populations that have not previously had it. In African countries with a high prevalence of the disease, improving health conditions would increase the life expectancy of people living with sickle cell disease and thus impose an additional burden on already precarious health systems (Piel et al., 2014; Martinez et al., 2014; Cataldo, 2012).

All these claims are based on mathematical models that make projections on the basis of poor-quality data. Adams (2016, p.6) demonstrated how the metrics aggregate distinct and disparate situations and contexts and then universalize these data, using them to promote health policies and programs, which are “imagined to offer uniform and standardized conversations about how best to intervene, how best to conceptualize health” (p.6). A recent publication by the Lancet Haematology Commission views the limited reliability, accuracy, and representativeness of data as a problem that hinders the production of reliable projections of the prevalence and distribution of sickle cell disease in most countries (Piel et al., 2023). This deficiency was detected in the report of the 2021 Global Burden of Disease, Injuries and Risk Factors Study, which overestimated the mortality rate of children under 5 years of age in France and the United Kingdom (Brousse et al., 2023) – countries where the data are supposedly more reliable than they are in low- and middle-income settings.

As the anthropologist Sangeeta Chattoo (2018, p.31; emphasis in the original) notes, there is a “a ‘crisis rhetoric’ of contagion in public health being extended to noncommunicable diseases across low- and middle-income countries.” There are already some studies that express criticism of the field of global health, demonstrating in particular how securitization and accountability can be oppressive and exacerbate preexisting inequities for vulnerable populations in the Global South (Reubi, Herrick, Brown, 2016; Biehl, 2011; Biehl, Petryna, 2013; Fan, Uretsky, 2017). These studies help us to understand how and why certain diseases become a priority, as is the case of noncommunicable diseases, which are highly dependent on the social determinants of health. Seeing them as diseases that are spread means ignoring the socio-environmental and political conditions that produce them.

Chattoo’s (2018) work has been pivotal in deepening the understanding that in the twenty-first century a global assemblage is taking shape that frames sickle cell disease as a threat to global health. This assemblage gained strength as of 2010, when the WHO reclassified the disease, transferring it from the group of inherited blood disorders to the “prevention and management of birth defects” group (WHO, 2010a). This change provides a stronger rationale for those who support reproductive control and population screening policies and for arguments in favor of preventing people with sickle cell disease from having children (Chattoo, 2018). Also in 2010, the WHO Regional Committee for Africa emphasized, first and foremost, the control of the disease through birth prevention, alluding to Millennium Development Goals 4 and 5, which concern the reduction of child mortality under the age of 5 and the improvement of maternal health (WHO, 2010b). Like Chattoo (2018), Fottrell and Osrin (2013) have demonstrated the perversity of correlating birth control among people with hemoglobinopathies and the Millennium Development Goals, even if it is done with the best of intentions, because birth control does not improve the health of that population, whereas accessible, well-structured health systems would.

Wherever there is a reproductive control policy, there will always be suspicions of eugenics, because policies like this go beyond the private and individual sphere to impose a specific conception of health. Prioritizing abortion, prevention, and reproductive control for people with sickle cell trait and disease has the effect of obscuring the value of comprehensive healthcare, which could include education and public communication campaigns and neonatal screening, as well as associated genetic counseling. As is the case in Brazil, even if its delivery is patchy, genetic counseling is part of the country’s comprehensive healthcare policy for people with sickle cell disease (Brasil, 2014).

The debate on the burden of sickle cell disease on global health is taking place amid urgent demands, such as the new wave of migration and growing pressures on public and state health systems due to the neoliberal trend towards the privatization of healthcare. Many leaders in the field of science are heavily committed to this privatizing and/or reductionist agenda, which is already being seen in countries such as Cuba, Cyprus, Italy, and India (Kountouris et al., 2016; Kato et al., 2018; Chattopadhyay, 2006; Diniz, Guedes, 2003). Nevertheless, it is generally accepted that the life expectancy of adults with sickle cell disease in high- and middle-income countries has been increased through continuous treatment, which is only possible in a comprehensive health system providing PHC (Kato et al., 2018, p.22; Brasil, 2014).

Furthermore, the sudden interest in this neglected disease is not the result of some humanitarian turn. Sickle cell disease remains neglected even in countries where it is prevalent (Chakravorty, Williams, 2015), which is a strong indication that “these are neglected diseases because they are of neglected people” (Oliveira, 2018, p.2300). It is not, therefore, the disease that is marginalized and subaltern, but the people and bodies in which it is manifested. From the perspective of necropolitics (Mbembe, 2016), disregard for people with sickle cell disease and the current drive to prevent their existence rather than providing proper (medical) care for them reveal how global health policies can be structured in an ungrounded manner. We are speaking of a chronic neglected disease that is said to threaten populations “free” of the gene, whose treatment would imply health expenditures beyond any country’s budget, regardless of its level of economic development and its existing health system.

Contemporary humanitarianism, which underpins many global health policies, ends up perpetuating violence and exacerbating social (Fassin, 2012) and racial (Thomas, Clarke, 2013) inequalities. In the case of sickle cell disease, the humanitarian argument for birth prevention hinges on children under 5 and their suffering, but fails to consider the millions of adults who suffer from lack of care (Chattoo, 2018). Instead of questioning the preexisting structures that have perpetuated the dearth of medical care for people with the disease, decision-makers have reached a solution (birth prevention) that reinforces the discourses of insecurity (dissemination/contagion) and accountability – two of the major pillars of global health. By so doing, they perpetuate structures of exclusion and control of these vulnerable populations.

Like Chattoo (2018), I am not suggesting we should understate the growth in sickle cell disease cases or reject prevention as a health strategy. Nonetheless, it behooves us to question both the intended scope and the priorities put on the table when a prevention policy is chosen for a disease that is reasonably well managed in contexts where there is minimal access to health services. The new geopolitical, economic, scientific, and cultural designs of neoliberalism are changing the medical and social interpretation of sickle cell disease.

The global assemblage taking shape around sickle cell disease in the twenty-first century is not historically isolated. In 1983, the WHO’s Community Control of Hereditary Anaemias working group issued a memorandum stating that successful examples of thalassemia control in countries such as Cyprus, Italy, Greece, the UK, and the USA could be disseminated and implemented globally (Boyo et al., 1983). It is beyond the scope of this article to elaborate on the social history of thalassemia, but it is worth pointing out that the beginnings of this global assemblage lie in the social contexts of very specific regions of Mediterranean Europe.

Not only do sickle cell disease and thalassemia have different symptoms, treatments, and prognoses, but the social history of thalassemia that has been documented is from European countries (Greece, Italy, Cyprus, and the UK) and the USA. Although studies have been done on its history in other regions in recent years, it is important to understand that the basis for health policymaking for thalassemia has been grounded (to use Burawoy’s expression) in these specific Mediterranean contexts. The history of thalassemia control in Cyprus, for example, is unique in this sense, since it was from an island context, with a population depleted by chronic emigration, among other factors, that global projections were made.

The WHO memorandum already recognized that for Africa, for example, where large families are the norm and abortion is widely rejected for cultural and religious reasons, “a simple approach to treatment is likely to be preferable to advanced methods of prevention culminating in mid-trimester abortion of affected fetuses” (Boyo et al., 1983, p.76). Furthermore,

the health burden of sickle cell disease is difficult to quantify because its natural history is so dependent on social conditions. ... It is debatable whether much effort should be deployed on sickle cell anaemia prevention programmes when relatively simple measures can lead to great improvement in the survival and quality of life of homozygotes [HbSS] (Boyo et al., 1983, p.66, 76).

The WHO’s Community Control of Hereditary Anaemias working group argued that in view of the cultural and biological differences between the two diseases, they should be addressed with different health policies (Boyo et al., 1983, p.70). There was also a crucial technical issue for those insular contexts – and one that weighed heavily when birth control measures were proposed. The argument went that frequent blood transfusions that are part of standard thalassemia treatment – but required only occasionally for sickle cell disease – would increase the demand for blood to such an extent that it would be impossible to obtain the necessary quantity in the future. In addition to disregarding the considerable differences between thalassemia and sickle cell disease, the movement in favor of global policies for hemoglobinopathy prevention also erases national and regional differences in the proportion of people with sickle cell trait and disease, cultural differences in the institution of marriage, the potential to have a good quality of life, and the ability of people to contribute to their country’s and region’s economies, among many other considerations.

In 1994, the WHO published GUIDELINES FOR THE CONTROL OF HAEMOGLOBIN DISORDERS, which reveals that thalassemia control programs did indeed serve as a model for recommending control measures for all hemoglobinopathies:

The best model for such services are the disease-orientated ‘thalassaemia control programmes’ organised in some countries of the mediterranean area where thalassaemia is particularly common ... Each country will need to develop an individual strategy appropriate to the local epidemiology, current service structure and available economic resources. WHO monitoring shows that it is easiest to organise an effective programme in a relatively small population where the disorders are common (WHO, 1994, p.3).

In 2006, the World Bank took a similar stance on the issue, suggesting two approaches to reducing the number of births “affected” with hemoglobinopathies – population screening and counseling; or population screening or screening in prenatal clinics, followed by abortion – which should be adopted according to the local context (Weatherall et al., 2006, p.668). The former approach did not work in Greece, since marriages were not affected by the genetic results, but it did work in Iran. Nonetheless, the World Bank adds that “data about the effectiveness of this approach are extremely limited” (p.669). The second strategy was adopted in different locations to target thalassemia. Thus, as in the documents already mentioned, it was understood that the approach for sickle cell disease should not be the same as that for thalassemia because it is “not uniformly fatal in early life, and morbidity and mortality during this period can be controlled,” and the “clinical course of the condition is unpredictable” (p.670).

Although genetics is important for public policymaking, changes that would affect individual lives and society at large cannot be made without drawing on knowledge from the human and social sciences. Our knowledge about the social determinants of health already shows that health problems will not be solved by genomics (and, now, precision medicine) unless social inequalities are tackled (Iriart, 2019). The Human Genome Project was designed with the aim of enabling the cure of genetic diseases, among other things. While it has achieved little in this regard, by the end of the twentieth century it had already contributed to establishing an interdisciplinary relationship among genomics, computing, and mathematics that has elevated bioinformatics to a prominent position in biomedical methodology (Salter, Salter, 2017).

Bioinformatics has made genomics marketable and manufacturable; as a result, it has become a scientifically productive discipline and has therefore garnered more political support (Stevens, 2011). In the last ten years, a supposed revolution seems to have emerged with the rise of precision medicine, which promises to include data beyond the genome, such as lifestyle and socio-environmental factors. These data are transformed into quantitative, digital inputs for databases, whose outputs are then used to make predictive analyses of the behavior of genes, mediated by environmental and lifestyle factors (Iriart, 2019, p.5).

Metrics, securitization, accountability, and evidence-based medicine are shaping global health in contemporary times and the health policies that best align with their quantifying principles. Since decision-making is based on diseases (or even on metrics generated about diseases), not on people, the international actors who finance global actions want quantifiable evidence, which ends up shaping the results that will appear as proof of public health conditions (Ventura, 2019; Fan, Uretsky, 2017; Erikson, 2016; Elbe, 2010; Adams, 2016). The most easily produced metrics for sickle cell disease are the numbers of individuals who will not be born with this disease, since accounting for the good quality of life of its carriers provided by a comprehensive care model would be time-consuming and would not yield returns on investments in global health. Prevention by reducing births is therefore more easily accounted for than caring for people with sickle cell disease, making it the most attractive policy option for the main promoters of global health.

### Neoliberalism and the international dissemination of the Unified Health System: expansion for resistance

Brazil has been one of the most attractive markets for private healthcare since the early 1900s, when foreign pharmaceutical companies competed for access to this, the second largest country in the Americas (Quintaneiro, 2002; Cavalcanti, Sá, 2017). Recent analyses show that private healthcare is undergoing a period of financialization through the concentration and expansion of its activities, which has a direct influence on the structure and functioning of SUS. This makes it all the more important to be able to identify “business agendas and the reception of corporate demands by government institutions” when developing and scrutinizing public policies (Bahia et al., 2022).

Since the 1990s, when it was introduced, SUS has been pivotal in the delivery of healthcare in the country. Nonetheless, it is an ongoing process, and one of its main difficulties is the relationship between the private sector and public spheres (Paim et al., 2011; Rodrigues, 2014). The World Bank has monitored this process in Brazil, producing regular publications, and, crucially, making loan agreements with states and municipalities, where there may be less resistance than at federal level. At these subnational levels, it is easier to execute programs aligned with the precepts of greater private sector participation, employment deregulation, and more focus on supposedly technical solutions unrestricted by political imperatives. As Rizzoto and Campos (2016, p.274) put it, “SUS is an experiment that, from the [World] Bank’s perspective, should not be expanded to other Latin American countries or even to the world.”

The defense of free, comprehensive healthcare is at the heart of SUS and its continued existence, which is threatened nationally and internationally by domestic and external pressures. The creation and rollout of SUS was a unique event globally in the 1990s because of the guiding principles of its participatory management model and the public right to health, and because it gave new life to a political strategy of social welfare that went into decline with the rise of neoliberalism (Paiva, Pires-Alves, 2011; Rodrigues, 2014). It is no coincidence that Sergio Arouca, one of the figureheads of the Brazilian health reform, in full awareness of this difficulty, prepared the draft of what would become the PRODIRS program in 2003, in a joint initiative of the Ministry of Health and the Pan American Health Organization (PAHO) (Paiva, Pires-Alves, 2011). In its guidelines, the program emphasized the need for international recognition of the Brazilian health policy and the possibility of technology and knowledge exchanges with other countries (Brasil, 2003). However, the program was never officially implemented.

In the last quarter of the twentieth century, traditional international cooperation in the health sector in Brazil began to be influenced by voices critical from the health reform movement (Almeida, 2017). By the dawn of the twenty-first century, the country’s model of technical cooperation in health was geared towards curbing the incursion of neoliberalism through “the institutional strengthening of the health systems of partner countries, combining concrete interventions with local capacity-building and knowledge generation ... in order to enable the countries themselves to take leadership of their health sector processes” (Almeida et al., 2010, p.28).

Camara (2023), who investigated Brazil’s technical cooperation in health with the Community of Portuguese-Speaking Countries (CPLP), points out that the materialization of technical cooperation in health is a consequence of efforts to expand SUS internationally, largely expressed in the ill-fated PRODIRS program. The defense of a proactive role for Brazil in cooperations that would strengthen the health systems of countries in the Americas and the CPLP, drawing on its experience in implementing SUS, appears in the Technical Cooperation Strategy for PAHO/WHO and the Federative Republic of Brazil, 2008-2012 (Paiva, Pires-Alves, 2011). At the turn of the twenty-first century, the global health prominence Brazil gained when it breached AIDS drug patent protections and introduced its HIV/AIDS program epitomizes the country’s bid to disseminate the principles of universalization and equality enshrined in its health reform and public health system (Cueto, Lopes, 2021). Structural technical cooperation in health in the context of sickle cell disease is also a facet of this stage in the international history of the Brazilian health reform.

### Structuring technical cooperation initiatives in health with Benin, Ghana, and Senegal

As mentioned at the beginning of this article, in late 2006, the coordinator of the national sickle cell disease policy (PNAIPDF), Doctor Joice Aragão de Jesus, spoke at the third Congress of the International Organization for the Fight against Sickle Cell Disease, in Dakar, sparking a controversy that prevented the signing of a memorandum of understanding that would have supported “education for prevention” as a priority policy for some African countries. The following day, Aragão de Jesus was called to the office of the first lady of Senegal, Viviane Wade, where she again explained how Brazil’s policy works within the framework of SUS (Jesus, Cortez, 14 maio 2024). It was from this point on that Brazil began to receive new requests for cooperation. ^2^

These requests arose amid the promotion of other types of technical cooperation in health between Brazil and Africa in the early 2000s, such as the Strategic Plan for Cooperation in Health (Plano Estratégico de Cooperação em Saúde, PECS) of CPLP, which started to take shape after the signing of the Estoril Declaration, in 2009 (Silva, Rosenberg, Fonseca, 2017, p.571-573). The PECS plan was for the development of health systems and, through them, the assurance of universal access to health through the capacity building and training of health workers and institutional strengthening through structuring projects. The idea of PECS was similar to the structuring cooperation in health that Brazil was beginning to promote in its bilateral agreements at the time. According to Almeida et al. (2010, p.28), this type of cooperation sought to build local capacity in order to strengthen existing health institutions, such as ministries of health, schools of public health, universities, and technology development institutes, and even contribute to the creation of new institutions.

The first request for cooperation in sickle cell disease came from Senegal as a result of both the aforementioned controversy and the work of Ambassador Katia Gilaberte, who was keen to convince the Senegalese government of the viability of the Brazilian comprehensive care model for people with sickle cell disease in Senegal. More broadly, in March 2007, the ministries of foreign affairs of both Brazil and Senegal signed the Oslo Declaration, which embraced health as a foreign policy issue, bringing them closer in their perspective on the role of health in geopolitics (Paiva, Pires-Alves, 2011).

In October 2007, Joice Aragão de Jesus returned to Senegal with a technical delegation from the Ministry of Health and the support of the Brazilian Cooperation Agency to give training in the model of care provided to people with sickle cell disease in Brazil. During this second visit, the Brazilian delegates realized that Senegal would need training to operate the neonatal screening equipment that had been donated by Canada. This visit was followed, in 2008, by the first visit of Senegalese technical personnel to Brazil. This seems to have been crucial for the signing of the official project document, since during this visit, the physician Ibrahima Diagne, responsible for the project’s guidelines, chose the PHC ^3^ activities carried out at the Martagão Gesteira Institute of Childcare and Pediatrics of the Federal University of Rio de Janeiro (UFRJ) as the most appropriate for the Senegalese context. It was the negotiations and interactions between Brazilian and Senegalese professionals during these visits that unthroned the conviction that a more restrictive health policy would be more appropriate for Senegal (Brasil, 2012, p.54).

This first experience with Senegal was what confirmed, for the Brazilian Ministry of Health professionals, that structuring projects for sickle cell disease could be carried out in countries with a high prevalence of the disease that did not have a free and universal health system, like SUS. Their enthusiasm also stemmed from the possibility of being able to undermine the belief that priority policies in low- and middle-income sub-Saharan countries should be directed towards the reproductive control of all individuals with sickle cell trait or disease. The Brazilians were well received by some key figures working to improve care for sickle cell disease in some sub-Saharan countries.

Structural technical cooperation for sickle cell disease was facilitated by transnational scientific relations between scientists and physicians specialized in the disease. The invitation for Brazil to participate in the Dakar conference, for example, was initially extended to the physician from the Pernambuco Hematology and Hemotherapy Foundation (Fundação de Hematologia e Hemoterapia de Pernambuco, Hemope), Doctor Aderson Araujo, who passed it on to the Brazilian Cooperation Agency because it was a meeting of first ladies. Before that, Brazilians had already participated in several international seminars besides the ones they organized in Brazil, such as the international symposia on hemoglobinopathies, which began in 2001.

The main player in Brazil’s cooperation with Ghana, for example, was the internationally renowned physician Kwaku Ohene-Frempong, who first visited Brazil in 2000 for the Brazilian Congress of Hematology, Hemotherapy and Cell Therapy, in Rio de Janeiro. During this visit, he met Clarice Lobo, who at the time was director of the Rio de Janeiro State Institute of Hematology (Hemorio), which already had a system for monitoring people with sickle cell disease, under the support of the State Program for Sickle Cell Anemia, created in 1998 and headed by Joice Aragão de Jesus until her transfer to the Ministry of Health, in 2004. In September 2007, when cooperation with Senegal was beginning, Ohene-Frempong, along with other foreign specialists, attended the fourth International Symposium on Hemoglobinopathies, in Rio de Janeiro, which was this time organized not by specialists but by the Ministry of Health (Lobo, 23 maio 2024).

In April 2008, specialists from the Ministry of Health made an official visit to research centers in the United States and met with the Ghanaian physician Ohene-Frempong, who drew on his connections in politics in Ghana to kick-started negotiations for cooperation in health for sickle cell disease with Brazil (Brasil, 2012). Another factor that boosted this cooperation was the involvement of the Brazilian Cooperation Agency in Ghana, which, since 2006, had helped develop technical cooperation in agriculture – the sector that received the most South-South cooperation investment (Brasil, 2011, p.40).

The first cooperation agreement was intended to help Ghana set up a nationwide comprehensive care system for people with sickle cell disease, which involved the “capacity building and training of Ghanaian professionals in a universal newborn screening program” (ABC, 2010, p.69). This agreement was quickly rolled out, as there was great interest in introducing a blood transfusion center specialized in the care of people with sickle cell disease to serve as a reference for the whole of West Africa (Brasil, 2012, p.71). The issue of blood quality was crucial in the cooperation negotiations, given its importance for the treatment of sickle cell disease.

These two cooperation projects with Ghana were planned between April and December 2009. The first was executed quickly and also involved actions in Brazil, specifically the training of Ghanaian technicians in neonatal screening at the Diagnostic Support and Research Unit (Núcleo de Ações e Pesquisa em Apoio Diagnóstico) of the School of Medicine at the Universidade Federal de Minas Gerais and the Minas Gerais State Foundation for Hematology and Hemotherapy, Hemominas, and the training of Ghanaian nurses in the comprehensive health care model offered by SUS (Brasil, 2012, p.70-73). Neonatal screening began in 1995 in Ghana, making it one of the first countries in Africa to provide this service. In ten years, more than two hundred thousand newborns were screened, with almost two percent of this total having sickle cell disease (Ohene-Frempong et al., 2008). However, there was no continuing education for health workers, which is why the cooperation with Brazil included capacity building.

During the conversations between Brazilians and the Ghanaians Kwaku Ohene-Frempong and Isaac Odame, based in the United States and Canada, respectively, there was great enthusiasm about few and critical resources that were available for children with sickle cell disease in Brazil, such as neonatal screening, penicillin and vaccines (Jesus, Cortez, 14 maio 2024; Araujo, 14 jun. 2024). Aderson Araujo reported that they were keen to understand how SUS worked, mentioning that, when attending an international conference, he had been invited by Isaac Odame himself to tell other specialists about the Brazilian experience in treating people with sickle cell disease (Araujo, 14 jun. 2024). Meanwhile, Odame remarked in publications on how Brazil served as a good example of a middle-income country that provided the minimum for the survival of children under 5 years old and offered hydroxyurea free of charge to a sizeable population of adults (Aygun, Odame, 2012).

Dialogue with Senegalese and Ghanaian stakeholders and negotiations for cooperation agreements were already advancing well in January 2009, when a delegation of Brazilian experts participated in the meeting Advancing Sickle Cell Disease Patient Care Through Global Research, in the capital of the Republic of Benin, Cotonou. The meeting, attended by people from 24 other countries, aimed to establish long-term partnerships and cooperation among high-, middle-, and low-income countries to “o further research and improve clinical care globally” (Odame, 2010, p.571). It is worth noting that none of the six working groups made any mention of population screening, premarital testing, selective abortions etc. Rather, they addressed the following points: neonatal screening, education, counseling, and care; infectious diseases in sickle cell disease; hydroxyurea therapy in Africa and other developing regions; a global approach to the genetic factors involved in phenotypic diversity; capacity building through a global sickle cell disease network and the development of an action plan; and discussions on experiences in collaborative projects to set up sickle cell disease centers in low-income countries (Odame, 2010). Although I cannot say that birth prevention was not discussed at the congress, it would seem that the subject was not as much of a priority as it had been at events such as the third Congress of the International Organization for the Fight against Sickle Cell Disease, in Dakar, in 2006, and in international publications, such as scientific articles and WHO documents.

At the event in Cotonou, the Brazilian group suggested making sickle cell disease a reason to structure health systems along the lines of SUS in African countries with a high prevalence of the disease. ^4^ This strategy would ensure there were health services available at the primary, secondary, and tertiary levels (Brasil, 2012, p.82). Taking advantage of the recently signed health cooperation agreement, the Brazilian group was invited to meet with the Beninese minister of health and the Brazilian ambassador to begin a project on sickle cell disease. Within two months, Benin and Brazil had signed the Sickle Cell Disease Pilot Project, through which Beninese health workers would receive training in Brazil – in neonatal screening at the Diagnostic Support and Research unit of the School of Medicine of UFMG and at Hemominas (p.82-83) – and Brazilian health workers would facilitate training in the use of the neonatal screening equipment received by Benin. Improvements in hemotherapy were also a priority due to the use of blood as a treatment, and were included in the cooperation agreement (Januário, 28 maio 2024).

Like Ghana, Benin had worked with neonatal screening since the 1990s (Rahimy et al., 2003). Between 1993 and 1999, Mohamed Cherif Rahimy, who headed this technical cooperation project with Brazil, conducted a study on infant monitoring in a comprehensive health care program, revealing its importance for reducing morbidity and mortality. Other important conclusions were the perception of the need to have trained and committed teams, and the finding that the program did not require significant resources and could therefore be implemented in places where recourses were in short supply (Rahimy et al., 2003, p.838). Although the lack of basic health structure is a major obstacle to implementing programs like this in sub-Saharan Africa, for this program the lack of medical education was one of the biggest hurdles, as it prevented diagnosis and subsequent monitoring, which are vital for the survival of children under 5 years of age.

It is important to highlight the role of Isaac Odame and Kwaku Ohene-Frempong on the international scene and their work in spreading the word about the Brazilian experience. In 2010, they both participated in the creation of the Global Sickle Cell Disease Network, ^5^ which held its first conference in 2011 in Accra, Ghana, and its second in 2014 in Rio de Janeiro, organized by Hemorio and the Ministry of Health department responsible for the coordination of blood and hemoderivatives. According to Odame, Kulkarni and Ohene-Frempong (2011, p.417), this network was created to enable the better representation of people with sickle cell disease, as was the case with the International Thalassemia Federation. The idea had first been raised at an event in November 2007 organized by this federation and the WHO, as set forth in MANAGEMENT OF HAEMOGLOBIN DISORDERS: REPORT OF A JOINT WHO-TIF MEETING.

Let us now return to the comparison made in the first section of this article between thalassemia and sickle cell disease. The existence of an international organization to advocate for people with sickle cell disease was essential for supporting general awareness and education about the disease. The mobilizing experience of the International Thalassemia Federation may continue to be an example, but given the huge differences between the diseases, despite both being hemoglobinopathies, it is debatable whether the priority policies should be the same.

Brazil’s structuring technical cooperation initiatives for sickle cell disease with Benin, Ghana, and Senegal showed that it was possible, even where resources were limited, to provide healthcare and monitoring for children born with sickle cell disease in their first years of life. The main actions of the programs focused on training for neonatal screening, which is the basic prerequisite for delivering any treatment and improving quality of life. According to the Brazilians involved in the programs, the reputation of Brazil’s vaccination and breastfeeding programs heightened interest in structural technical partnerships in health for sickle cell disease (Jesus, Cortez, 14 maio 2024; Januário, 28 maio 2024).

## Final considerations

Brazil’s prioritization of comprehensive care for people with sickle cell disease, rather than reproductive control, reveals not only an ethical stance vis-a-vis people with the trait and disease, but also a degree of political opportunities in the context of South-South cooperation at a time when Brazil was reaching out to developing countries to strengthen its own international standing. The design of a global policy for the prevention of sickle cell disease in the twenty-first century occurred at the same time as technical cooperation initiatives in health that foregrounded the Brazilian policy for people with the disease, which only worked due to the existence of SUS. With this and other health cooperation initiatives, Brazil wanted to spread its PHC model (Almeida et al., 2010) and, in the case of sickle cell disease, to oppose the birth prevention policy. The funds invested jointly by the Ministry of Foreign Affairs and the Ministry of Health in this cooperation for sickle cell disease were not inconsiderable, coming second only the investments made in HIV/AIDS-related projects (Tagliari, 2014). ^6^

The demonstration by Brazil that it was possible for a middle-income country to care for its sickle cell population was important in raising awareness in countries such as Benin, Ghana, and Senegal, which, despite having had some experience in this regard, did not know how these people were monitored in Brazil by SUS. The issue of the resources needed to treat people with sickle cell disease is always presented in an oversimplified, as if all contexts were the same. Brazil showed that it is possible to carry out adequate monitoring, provided there is a structured health system in place (even if it is suboptimal), and that this monitoring reduces the costs of the disease by addressing complications early. As Joice Aragão de Jesus explains, the costs of treating people with sickle cell disease are embedded in SUS, meaning that they are shared amongst thousands of other users with different diseases and health problems, because there is no single specific treatment, exam, or procedure for sickle cell disease (Jesus, Cortez, 14 maio 2024).

Brazil’s technical cooperation effort in health was embedded in various other unfolding historical processes in the arenas of global health (consolidation of the primacy of metrics, securitization, and accountability), national health policy (consolidation of a free and universal health system), and the long history of the medical/scientific interpretation of sickle cell disease (changing the definition from an inherited blood disease to a preventable birth defect). In one sphere of the international arena, where the WHO and other international organizations work in partnership with nation-states, policies to control populations with sickle cell trait and disease have been promoted, along with acknowledgements of the importance of access to healthcare for people with the disease. In another sphere, we have seen the dissemination of the Brazilian public health service model offered to people with sickle cell disease in a bid to spread and exchange the values of universalism and equality enshrined in the Brazilian health reform and its embodiment, SUS.

## Data Availability

Not deposited in a data repository.

## NOTES

1 Ethical approval for the four interviews conducted for this research was received on November 16, 2023, from the research ethics committee of Escola Politécnica de Saúde Joaquim Venâncio/Fiocruz (approval number 73202323.6.0000.5241).

2 PHC here refers to activities to prevent complications in care when newborns are diagnosed with the disease and the promotion of self-care in adults.

3 The members of the Brazilian group were: Anna Bárbara Proeitti (Hemominas), Aderson Araujo (Hemope), Clarice Lobo (Hemorio), Guilherme Genovez (CGSH), Joice Aragão de Jesus (PNAIPDF), José Nélio Januário (Nupad/UFMG), Marilda Gonçalves (Fiocruz/Bahia), and Paulo Ivo Cortez (UFRJ).

4 The members of the Brazilian group were: Anna Bárbara Proeitti (Hemominas), Aderson Araujo (Hemope), Clarice Lobo (Hemorio), Guilherme Genovez (CGSH), Joice Aragão de Jesus (PNAIPDF), José Nélio Januário (Nupad/UFMG), Marilda Gonçalves (Fiocruz/Bahia), and Paulo Ivo Cortez (UFRJ).

5 Available at: https://www.globalsicklecelldisease.org/about-sickle-cell-disease . Access on: 2 out. 2024.

6 While it may seem as if investment in technical cooperation for sickle cell disease was focused simply on the disease (Tagliari, 2014), as was the case for HIV/AIDS, in fact it branched out into training for neonatal screening and improving the blood supply; i.e., improving the basic health service (Brasil, 2012; Januário, 28 maio 2024).
