# Supplementary material for: International dissemination of Brazil’s public health system and its technical cooperation initiatives for sickle cell disease, 2006-2010
Source: Hist Cienc Saude Manguinhos. 2025 Sep 22;32:e2025039. doi: 10.1590/S0104-59702025000100039en (PMC12459757; doi:10.1590/S0104-59702025000100039en)
Supplement: Translation

# Translation: A divulgação internacional do Sistema Único de Saúde e as cooperações técnicas em saúde para a doença falciforme, 2006-2010

**DOI:** 10.1590/S0104-59702025000100039

**Authors:** Juliana Manzoni Cavalcanti

**Affiliations:** i Pesquisadora,Casa de Oswaldo Cruz/Fiocruz Rio de Janeiro – RJ – Brasil juliana.manzoni@fiocruz.br

**Keywords:** Brasil, Doença falciforme, Cooperação Sul-Sul, Saúde global, Sistema Único de Saúde (SUS).

## Abstract

Este trabalho se alinha a estudos que buscam analisar criticamente a constituição da doença falciforme como ônus para a saúde global a partir dos anos 2000. Para isso, investigam-se as tentativas de cooperação técnica estruturante em saúde do Brasil, no âmbito do modelo de atenção integral à saúde ofertado às pessoas com doença falciforme no Sistema Único de Saúde (SUS), em Benim, Gana e Senegal. Essa atuação internacional é uma amostra de iniciativas à disseminação internacional dos valores universalistas e igualitários da reforma sanitária brasileira presentes no SUS.

## ANÁLISE

No final de 2006, a médica Joice Aragão de Jesus apresentou, no terceiro Congresso da Organização Internacional de Combate à Doença Falciforme, o modelo de atenção integral à saúde ofertado às pessoas com doença falciforme no âmbito do Sistema Único de Saúde (SUS). O evento, organizado pelas primeiras-damas da União Africana, tinha por objetivo chegar a consenso sobre uma política global prioritária para a doença falciforme, que já se materializava por meio de diferentes documentos da Organização Mundial da Saúde (OMS) e da Organização das Nações Unidas para a Educação, a Ciência e a Cultura (Unesco) na direção do controle de nascimentos (Unesco, 2005; WHO, 2006, 2010a).

Joice Aragão de Jesus, que, havia cerca de dois anos, comandava a Política Nacional de Atenção Integral às Pessoas com Doença Falciforme e Outras Hemoglobinopatias (PNAIPDF), causou polêmica quando explicou que o Brasil investia no cuidado integral da pessoa com falciforme, e não em uma política de controle reprodutivo. A fala da médica brasileira suspendeu a assinatura do memorando de entendimento em prol do controle reprodutivo de pessoas com traço e doença falciformes (Jesus, Cortez, 14 maio 2024). A partir de então, o Brasil, por intermédio de representantes da PNAIPDF, do Ministério da Saúde e da Agência Brasileira de Cooperação do Ministério das Relações Exteriores, empreendeu viagens por países da África subsaariana e Jamaica para apresentar o modelo de atenção integral à saúde ofertado às pessoas com doença falciforme no SUS (Brasil, 2012).

O objetivo deste artigo é contribuir para os estudos que vêm analisando criticamente a ascensão da doença falciforme como um ônus para a saúde global no século XXI. Para isso, tenho investigado algumas das tentativas de cooperação técnica em saúde do Brasil no âmbito do modelo de atenção integral à saúde disponível às pessoas com doença falciforme no SUS com nações subsaarianas com alta prevalência da doença. A atenção integral à saúde consiste na prevenção, promoção, assistência e recuperação da saúde, levando em conta o conceito de integralidade da pessoa. Com isso, o indivíduo deve ser compreendido em seu meio histórico, político, social e familiar, ao longo de um acompanhamento vitalício, que se inicia com a Atenção Primária à Saúde (APS) e se desdobra em outros dois níveis de complexidade da atenção e dos serviços de saúde (Hartz, Contandriopoulos, 2004).

No contexto nacional, a política externa brasileira imprimia novo fôlego às Cooperações Sul-Sul com vistas ao fortalecimento do Brasil em um mundo multipolar (Almeida, 2017). As cooperações analisadas neste artigo, do Brasil com Benim, Gana e Senegal entre 2006 e 2010, podem também ser consideradas iniciativas de disseminação internacional dos valores universalistas e igualitários da reforma sanitária brasileira que regem institucionalmente o SUS.

No mesmo período, montava-se um enquadramento global (global assemblage framing), com o suporte de cientistas, gestores, associações de pacientes e a OMS, que definia a doença falciforme e as demais hemoglobinopatias como defeitos de nascença evitáveis (Chattoo, 2018). A genética tem sido a principal porta-voz do controle social e reprodutivo das hemoglobinopatias. Ao exemplificar, por exemplo, os casos de talassemia relativos a Chipre e Sardenha, que têm populações pequenas e conduzem políticas de prevenção há décadas, geneticistas projetam tais contextos para realidades muito mais complexas e amplas, como a de países como a Índia (Chattopadhyay, 2006). É notório que essa interpretação mais reducionista está sobressaindo no debate sobre saúde global em detrimento de uma compreensão mais ampla, que se apoia na importância da APS e no acompanhamento integral para cuidar das pessoas que têm hemoglobinopatias.

Analisar essa nova tendência da genética em relação às hemoglobinopatias requer investigações dos contextos locais nos quais o discurso do controle reprodutivo ressoa ou é rechaçado. Seguindo a noção de aterramento da globalização proposta por Burawoy et al. (2000) e a perspectiva posta por Biehl e Petryna (2013) de centralizar as pessoas nos estudos sobre políticas globais, analiso o papel de certos atores da política externa brasileira em saúde e o novo enquadramento médico e social da doença falciforme de inícios do século XXI para entender as cooperações do Brasil com Benim, Gana e Senegal entre 2006 e 2010. Mediante a história oral ^1^ e o exame de documentos, mostro como o modelo brasileiro de atenção integral à saúde ofertado pelo SUS às pessoas com doença falciforme pode abalar um almejado consenso relativo à adoção de uma política de saúde restritiva para pessoas com traço e doença falciformes e, ao mesmo tempo, contribuir para a disseminação dos valores da reforma sanitária brasileira, como a universalidade e a equidade.

O artigo está divido em três seções. Na primeira, com suporte da bibliografia, em especial do trabalho de Chattoo (2018), e analisando fontes da OMS e artigos científicos, monto breve panorama do surgimento desse novo enquadramento da doença falciforme como ameaça à saúde global. Na segunda seção, trato da disseminação e divulgação internacional dos princípios de equidade e da universalidade da reforma sanitária que estão legalmente representados pelo SUS, e o faço mediante bibliografia e o exame do malogrado Programa de Difusão e Intercâmbio sobre a Reforma Sanitária (Prodirs). Na terceira seção finalizo a argumentação com as cooperações técnicas em saúde realizadas de 2006 a 2010 entre Brasil, Benim, Gana e Senegal. Insiro essas iniciativas no histórico da cooperação estruturante brasileira em saúde no início do século XXI, respaldada pelas Cooperações Sul-Sul, e demonstro como a atuação do Brasil foi importante para pôr em debate ou em execução uma nova estratégia de saúde que redirecionou naqueles países o foco da doença falciforme para as pessoas por ela acometidas e, com isso, contribuiu para a valorização da atenção integral como estratégia de saúde.

### Políticas globais para a doença falciforme, genética e Organização Mundial da Saúde

A doença falciforme, desde o início do século XXI, tem sido considerada um ônus e ameaça global. Argumenta-se que o aumento da imigração para a Europa traz muitos indivíduos portadores da doença e do traço falciformes, e que, portanto, acarreta um pesado ônus a sistemas de saúde europeus, além de disseminar, segundo Weatherall e Clegg (2001) e Weatherall (2007), o gene entre populações que não o apresentam. Nos países africanos com alta prevalência, preconiza-se que a melhoria das condições de saúde aumentará a expectativa de vida das pessoas que vivem com a doença falciforme, o que levará a mais uma sobrecarga ao já precário sistema de saúde (Piel et al., 2014; Martinez et al., 2014; Cataldo, 2012).

Todas essas afirmações são feitas com base em modelos matemáticos que estabelecem projeções a partir de dados considerados de baixa confiabilidade. Adams (2016, p.6) demonstrou como as métricas agregam situações e contextos distintos e díspares em dados que se tornam universais, e, a partir deles, políticas e programas de saúde são projetados por meio de diálogos e conversas “padronizados sobre como melhor intervir, como melhor conceituar saúde” (p.6). Uma publicação recente da Lancet Haematology Commission destaca as baixas confiabilidade, precisão e representatividade dos dados como problemas que impedem a produção de projeções confiáveis da prevalência e da distribuição da doença falciforme na maioria dos países (Piel et al., 2023). Essa deficiência foi detectada no relatório do Global Burden of Disease, Injuries and Risk Factors Study, de 2021, que superestimou a taxa de mortalidade de crianças abaixo de 5 anos na França e no Reino Unido (Brousse et al., 2023), países em que os dados são supostamente mais confiáveis do que naqueles de renda baixa e média.

Como destacado pela antropóloga Sangeeta Chattoo (2018, p.31; destaque no original), “há uma ‘crise de retórica’ no conceito de contágio na saúde pública que está se estendendo às doenças não comunicáveis nos países de renda baixa e média”. Já temos alguns estudos críticos ao campo da saúde global, em especial aqueles que demonstram como a securitização e a accountability (prestação de contas) podem ser opressoras e exacerbar iniquidades preexistentes às populações vulneráveis do Sul Global (Reubi, Herrick, Brown, 2016; Biehl, 2011; Biehl, Petryna, 2013; Fan, Uretsky, 2017). Esses estudos nos levam ao entendimento de como e por que certas doenças ascendem ao patamar de prioridades, como, por exemplo, as doenças não comunicáveis (DNC), enfermidades muito dependentes dos determinantes sociais da saúde. Enxergá-las como doenças que se disseminam é ignorar os condicionantes socioambientais e políticos que as produzem.

O trabalho de Chattoo (2018) foi primordial para aprofundar o entendimento de que no século XXI se forma uma montagem global interpretativa (global assemblage framing) que classifica a doença falciforme como uma ameaça à saúde global. Tal montagem adquire densidade a partir de 2010, quando a OMS a reclassificou, transferindo-a do grupo de doença hereditária do sangue para o grupo de “prevenção e manejo de defeitos congênitos” (WHO, 2010a). Essa mudança conferiu melhor fundamentação aos promotores de políticas de controle reprodutivo e de triagem da população, que defendem a prevenção de nascimentos de pessoas com doença falciforme (Chattoo, 2018). Também em 2010, o Comitê Regional da OMS para a África reforça, em primeiro plano, o controle da doença pela prevenção de nascimentos, fazendo alusão aos objetivos 4 e 5 de desenvolvimento do milênio, que se referem à diminuição da mortalidade infantil abaixo dos 5 anos e à melhoria da saúde materna (WHO, 2010b). Fottrell e Osrin (2013), bem como Chattoo (2018), mostraram que a relação entre o controle de nascimentos no segmento de portadores das hemoglobinopatias e os objetivos de desenvolvimento do milênio pode ser perversa, mesmo que oriunda das melhores intenções, já que o controle de nascimentos não melhora a situação de saúde daquela população, ao contrário de um sistema de saúde acessível e bem estruturado.

Políticas de controle reprodutivo sempre estarão sob suspeita de ser medidas eugênicas porque ultrapassam o âmbito privado e individual, forçando uma determinada concepção de saúde. Ao priorizar o aborto, a prevenção e o controle reprodutivo de pessoas com o traço e a doença falciformes, obscurece-se o valor da atenção integral à saúde, pois nela pode haver campanhas de conscientização e educação e triagem neonatal, bem como o consequente aconselhamento genético. Como ocorre no caso do Brasil, ainda que nem sempre de maneira eficaz, inserido na política nacional está o aconselhamento genético, que é apenas parte de uma política de atenção integral à saúde para a pessoa com doença falciforme (Brasil, 2014).

O debate sobre o ônus da doença falciforme para a saúde global ocorre em meio a um contexto de demandas postas como urgentes, como a nova onda migratória e a crescente pressão sobre sistemas públicos e estatais de saúde pela tendência privatista do neoliberalismo. Há forte engajamento de lideranças científicas a essa agenda privatista e/ou reducionista, que já está em curso em países como Cuba, Chipre, Itália e Índia (Kountouris et al., 2016; Kato et al., 2018; Chattopadhyay, 2006; Diniz, Guedes, 2003). Há, porém, consenso quanto ao aumento da expectativa de vida de adultos com doença falciforme em países de alta e média renda ter sido alcançado pelo tratamento contínuo, possível apenas em um sistema de saúde primário e integral (Kato et al., 2018, p.22; Brasil, 2014).

Ademais, o repentino interesse em uma doença negligenciada não é consequência de uma reviravolta humanitária. A doença falciforme continua negligenciada até nos países com alta prevalência (Chakravorty, Williams, 2015), o que é forte indício de que “são doenças negligenciadas, pois que de pessoas negligenciadas” (Oliveira, 2018, p.2300). Não é, portanto, a doença que é marginalizada e subalterna, mas as pessoas e os corpos nos quais ela se expressa. Sob as lentes da necropolítica (Mbembe, 2016), o descaso para com as pessoas com doença falciforme e a atual mobilização para evitar sua existência, antes que haja condições dignas de atenção e assistência médica, revelam como as políticas globais de saúde podem ser estruturadas de maneira desaterrada. É uma doença negligenciada, de curso crônico, que se diz ameaçar as populações “livres” do gene, e que demanda gastos com saúde avaliados como inviáveis para quaisquer países, independentemente do grau de desenvolvimento econômico e do sistema de saúde disponível.

O humanitarismo contemporâneo, que está por trás de várias políticas de saúde global, acaba por perpetuar a violência e aguçar desigualdades sociais (Fassin, 2012) e raciais (Thomas, Clarke, 2013). No caso da doença falciforme, foram as crianças com menos de 5 anos e o seu sofrimento usados como motivo humanitário para a prevenção de nascimentos, esquecendo-se dos milhões de adultos que padecem da falta de cuidados (Chattoo, 2018). Em vez de questionar antigas estruturas que eternizam a falta de atenção médica à doença, preferiu-se criar uma solução (prevenção de nascimentos) que desse aval aos discursos da insegurança (disseminação/contágio) e da accountability, grandes sustentáculos na saúde global. Perpetuam-se, assim, estruturas de exclusão e controle dessas populações vulneráveis.

Não é o caso, seguindo o posicionamento de Chattoo (2018), de minimizar o aumento dos casos de doença falciforme, nem de detratar a prevenção como uma estratégia de saúde. É preciso, contudo, questionar tanto o escopo quanto as prioridades postas na mesa quando se elegem políticas de prevenção para uma doença que na atualidade é razoavelmente administrada, quando se tem um mínimo de acesso à saúde. O novo desenho geopolítico, econômico, científico e cultural do neoliberalismo está mudando a interpretação médica e social sobre a doença falciforme.

A montagem desse enquadramento global para a doença falciforme no século XXI tem história. Em 1983, o grupo de trabalho Community Control of Hereditary Anaemias, da OMS, afirmava em um memorando que exemplos bem-sucedidos de controle da talassemia em países como República do Chipre, Itália, Grécia, Reino Unido e EUA poderiam ser disseminados e implementados globalmente (Boyo et al., 1983). Não será o caso de aprofundar a história social da talassemia, mas é preciso indicar que o início desse enquadramento global repousa em contextos sociais de regiões da Europa Mediterrânea muito particulares.

Além de a doença falciforme e a talassemia diferirem em termos de sintomas, tratamentos e prognósticos, a história social da talassemia que tem sido documentada pertence ao contexto de países europeus (Grécia, Itália, Chipre e Reino Unido) e dos EUA. Embora avultem nos últimos anos os trabalhos sobre as demais regiões, o importante é entender que a base para a formulação de políticas de saúde para a talassemia se aterra (para usar a expressão de Burawoy) nesses contextos mediterrâneos específicos. A história do controle da talassemia em Chipre, por exemplo, é singular nesse sentido, pois é a partir de um contexto insular, de população reduzida especialmente pela emigração crônica, que se fizeram projeções mundiais.

No memorando da OMS já se reconhecia que, por exemplo, para a África, onde a norma são as grandes famílias e há muita rejeição ao aborto por questões culturais e religiosas, “uma abordagem simples de tratamento é possivelmente preferível aos métodos de prevenção avançados que culminam no aborto de fetos acometidos pela doença” (Boyo et al., 1983, p.76). Além disso,

o ônus da doença falciforme [aos sistemas de] saúde é difícil de quantificar porque sua história natural é muito dependente das condições sociais ... É discutível se devemos dispender muito esforço na implementação de programas de prevenção da doença falciforme quando medidas relativamente simples podem levar a um ótimo incremento na sobrevivência e na qualidade de vida dos homozigotos [HbSS] (Boyo et al., 1983, p.66, 76).

Argumentava-se que a diferença entre ambas as enfermidades, tanto cultural quanto biologicamente, deveria levar a distintas formulações de políticas de saúde (Boyo et al., 1983, p.70). Havia ainda uma questão técnica crucial para aqueles contextos insulares – que pesou na hora de propor medidas de controle de nascimentos. Afirmava-se que as transfusões frequentes, que fazem parte do protocolo de tratamento da talassemia, mas apenas eventualmente da falciforme, aumentariam a demanda por sangue de tal maneira que no futuro seria inviável obter a quantidade necessária. Além das consideráveis diferenças entre as talassemias e as doenças falciformes, o movimento em prol de políticas globais de prevenção das hemoglobinopatias apaga, portanto, as diferenças nacionais e regionais no que tangem à proporção de pessoas com traço e doença falciformes, às diferenças culturais na instituição do casamento, à possibilidade de viver bem, com qualidade, e contribuindo para o aumento do PIB de seu país/região, além de muitos outros aspectos.

Em 1994, a OMS divulgou o Guidelines for the control of haemoglobin disorders (Guia para o controle das desordens da hemoglobina), no qual constatamos que os programas de controle da talassemia realmente foram usados como modelo para sugerir medidas de controle a todas as hemoglobinopatias:

Para registrar, o melhor modelo de tais serviços [relativos às hemoglobinopatias] são os ‘programas de controle da talassemia’, orientados para a doença, em alguns países da área do Mediterrâneo onde a talassemia é particularmente comum ... Cada país deverá desenvolver uma estratégia individual apropriada à epidemiologia local, às estruturas dos serviços existentes e aos recursos econômicos disponíveis. A observação da OMS mostra que é muito mais fácil organizar um programa eficaz em uma população relativamente pequena em que as doenças são comuns (WHO, 1994, p.3).

Posicionando-se similarmente sobre a questão, em 2006, o Banco Mundial sugeriu para a redução de “nascidos afetados” pelas hemoglobinopatias duas abordagens (triagem e aconselhamento populacional; triagem populacional e em clínicas pré-natais com o aborto consequente) que deveriam ser adotadas a depender do contexto local (Weatherall et al., 2006, p.668). A primeira não deu certo na Grécia, pois os casamentos não foram afetados pelos resultados genéticos, mas teve sucesso no Irã. E o Banco Mundial completa que “os dados sobre a efetividade dessa abordagem são extremamente limitados” (p.669). A segunda estratégia foi abraçada em diferentes locais visando à talassemia. Assim, como nos documentos já mencionados, interpretava-se que a doença falciforme não deveria ser encarada tal como a talassemia porque ela não é “uniformemente fatal em tenra idade, e a morbidade e mortalidade durante esse período podem ser controladas ... [além de] o curso clínico da condição ser imprevisível” (p.670).

Apesar de a genética ser ferramenta necessária e importante na definição de políticas públicas, ela não pode prescindir dos conhecimentos das ciências humanas que lhe deem respaldo para sugerir mudanças na vida individual e no corpo social. Os conhecimentos que temos sobre os determinantes sociais da saúde já nos mostram que a genômica e agora a medicina da precisão não resolverão os problemas de saúde sem que as desigualdades sociais diminuam (Iriart, 2019). O Projeto Genoma Humano foi idealizado para, entre vários objetivos, viabilizar a cura de doenças genéticas. Pouco logrou, no entanto, nesse sentido, embora tenha contribuído para estabelecer entre a genômica, a computação e a matemática uma relação interdisciplinar que ascendeu a bioinformática a uma posição de destaque na metodologia da biomedicina ao final do século XX (Salter, Salter, 2017).

A bioinformática tornou a genômica industrializável e comercializável e, com isso, transformou-se em disciplina cientificamente produtiva e, por consequência, mais bem-sucedida politicamente (Stevens, 2011). Nos últimos dez anos, uma suposta revolução parece surgir com a medicina da precisão que traz, justamente, dados para além do genoma, como estilo de vida e condições socioambientais. É preciso frisar que tais dados continuam quantitativos, computadorizadas e digitalizados, pois servem para suprir bases de dados que supostamente alimentarão análises preditivas sobre o comportamento de genes, mediados por fatores ambientais e do estilo de vida (Iriart, 2019, p.5).

As métricas, a securitização, a accountability e a medicina baseada em evidências conformam a saúde global na contemporaneidade e determinam as políticas de saúde que melhor se aproximam de seus princípios quantificadores. Como a tomada de decisões é baseada em doenças (ou ainda nas métricas geradas sobre as doenças), e não nas pessoas, os atores internacionais que financiam as ações globais solicitam evidências contábeis, o que acaba moldando o resultado que aparecerá como prova das condições sanitárias (Ventura, 2019; Fan, Uretsky, 2017; Erikson, 2016; Elbe, 2010; Adams, 2016). As métricas produzidas mais facilmente para a doença falciforme são os números daqueles que não nascerão com essa enfermidade, pois contabilizar a boa qualidade de vida de seus portadores proporcionada em um modelo de atenção integral à saúde requer tempo e não gera rendimentos aos investimentos em saúde global. A prevenção mediante a diminuição de nascimentos é, por conseguinte, a solução mais facilmente contabilizada do que o cuidado das pessoas com doença falciforme, e, assim, torna-se a política que mais se enquadra nos interesses dos principais promotores da saúde global.

### Neoliberalismo e disseminação internacional do Sistema Único de Saúde: expandir para resistir

O Brasil é um dos países mais atrativos ao mercado privado da saúde desde inícios do século XX, quando empresas estrangeiras de medicamentos disputavam o mercado farmacêutico brasileiro, o segundo das Américas (Quintaneiro, 2002; Cavalcanti, Sá, 2017). Análises recentes mostram que o setor privado da saúde está passando por um momento de financeirização por meio da concentração e expansão de suas atividades que influenciam diretamente a estrutura e o funcionamento do SUS; portanto, saber identificar “agendas empresariais e recepção das demandas corporativas pelas instituições governamentais” é de suma importância para erigir e questionar políticas públicas (Bahia et al., 2022).

A partir dos anos 1990, a implementação do SUS é um marco na oferta de assistência à saúde no país, pois é um processo ainda em curso e tem como uma de suas principais dificuldades a relação entre o setor privado e as esferas públicas (Paim et al., 2011; Rodrigues, 2014). O Banco Mundial tem acompanhado esse processo no Brasil por meio de publicações, mas principalmente pelos contratos de empréstimos com estados e municípios, nos quais as resistências podem ser menores do que em nível nacional. Nesse âmbito fica mais fácil implementar programas alinhados às diretrizes de mais participação do setor privado, mais flexibilização das relações de trabalho e mais enfoque em soluções supostamente técnicas, livres de comprometimento político. O “SUS é um experimento que, na perspectiva do Banco [Mundial], não deve se expandir para outros países da América Latina e mesmo para o mundo” (Rizzoto, Campos, 2016, p.274).

A defesa da atenção integral e gratuita à saúde está no bojo da sustentabilidade e existência do SUS, ameaçado nacional e internacionalmente por pressões internas e externas. A criação e implementação do SUS na década de 1990 foi um evento isolado internacionalmente no que se refere aos seus princípios norteadores de gestão participativa e ao direito público à saúde, bem como por dar novo fôlego a uma estratégia política de bem-estar social que entrava em declínio com a ascensão do neoliberalismo (Paiva, Pires-Alves, 2011; Rodrigues, 2014). Não à toa, Sergio Arouca, eminente ator da reforma sanitária brasileira, ciente dessa dificuldade, elaborou em 2003 o rascunho do que seria o Prodirs, numa iniciativa conjunta do Ministério da Saúde com a Organização Pan-Americana da Saúde (Opas) (Paiva, Pires-Alves, 2011). Nas linhas desse programa, enfatizava-se a necessidade do reconhecimento internacional do modelo de política de saúde brasileiro e as possibilidades de intercâmbio de tecnologias e conhecimentos com outros países (Brasil, 2003). O programa, contudo, nunca foi oficialmente implementado.

No último quartel do século XX, a tradicional cooperação internacional no setor de saúde no Brasil passou a vivenciar as vozes críticas do movimento de reforma sanitária (Almeida, 2017). No alvorecer do século XXI, a cooperação técnica em saúde abraçada pelo país tinha como diretriz refrear o avanço do neoliberalismo mediante “o fortalecimento institucional dos sistemas de saúde dos países parceiros, combinando intervenções concretas com a construção de capacidades locais e a geração de conhecimento ... de forma a possibilitar que os próprios países assumam o protagonismo na liderança dos processos no setor saúde” (Almeida et al., 2010, p.28).

Ao investigar a cooperação técnica em saúde do Brasil com a Comunidade dos Países de Língua Portuguesa (CPLP), Camara (2023) aponta que a materialização da cooperação técnica em saúde é consequência dos esforços de expansão internacional do SUS, externados em linhas gerais no malogrado Prodirs. A defesa de uma atuação proativa do Brasil em cooperações que fortalecessem sistemas de saúde de países das Américas e da CPLP utilizando sua experiência na implementação do SUS aparece na Estratégia de Cooperação Técnica da Opas/OMS com a República Federativa do Brasil, 2008-2012 (Paiva, Pires-Alves, 2011). Na virada para o século XXI, o destaque do Brasil na saúde global com a quebra das patentes e o seu programa de combate à aids constituem-se em exemplos de divulgação dos princípios universalizantes e igualitários da reforma sanitária brasileira e do SUS (Cueto, Lopes, 2021). A cooperação técnica estruturante em saúde no âmbito da doença falciforme também se enquadra nesse capítulo da história internacional da reforma sanitária brasileira.

### A cooperação técnica estruturante em saúde do Brasil com Benim, Gana e Senegal

Como mencionado no início deste artigo, ao final de 2006, a coordenadora da PNAIPDF, doutora Joice Aragão de Jesus, fomentou, no terceiro Congresso da Organização Internacional de Combate à Doença Falciforme, em Dacar, uma polêmica que evitou a assinatura do memorando de entendimento que elegia a “Educação para prevenção” como política prioritária para alguns países da África. No dia seguinte, chamada ao escritório da primeira-dama do Senegal, Viviane Wade, Joice Aragão de Jesus voltou a explicar o funcionamento da política nacional sob as estruturas do SUS (Jesus, Cortez, 14 maio 2024). E foi a partir desse evento que começaram a surgir demandas ao Brasil por cooperação. ^2^

Tais demandas surgiram em meio à construção de outros tipos de cooperações técnicas em saúde entre Brasil e África no início do século XXI, como, por exemplo, as oriundas do Plano Estratégico de Cooperação em Saúde (Pecs) da CPLP, que começaram a se materializar após a assinatura da Declaração de Estoril em 2009 (Silva, Rosenberg, Fonseca, 2017, p.571-573). O Pecs visava ao desenvolvimento dos sistemas de saúde e, com isso, garantir o acesso universal à saúde mediante a capacitação e formação profissionais de saúde e o fortalecimento institucional por meio de projetos estruturantes. A ideia do Pecs era similar à denominada cooperação estruturante em saúde, que o Brasil passou a promover em seus acordos bilaterais no período. Na formulação de Almeida et al. (2010, p.28), esse tipo de cooperação buscava promover a capacitação local para que essa fortalecesse instituições de saúde já existentes, como ministérios da saúde, escolas de saúde pública, universidades, institutos de desenvolvimento tecnológico, ou mesmo que contribuísse para a criação de novas instituições.

A primeira demanda por cooperação em saúde para a doença falciforme veio do Senegal, em consequência da mencionada polêmica, mas também pela atuação da embaixadora Katia Gilaberte – que se empenhou no convencimento da viabilidade do modelo de atenção integral à saúde brasileiro às pessoas com doença falciforme no contexto senegalês. Numa angular mais ampla, em março de 2007, os Ministérios das Relações Exteriores do Brasil e do Senegal haviam assinado a Declaração de Oslo, na qual se abraçava a saúde como tema de política externa, aproximando-os quanto ao papel da saúde na geopolítica (Paiva, Pires-Alves, 2011).

Em outubro de 2007, Joice Aragão de Jesus voltava àquele país com uma comitiva de técnicos do Ministério da Saúde e com o respaldo da Agência Brasileira de Cooperação, para promover a capacitação local no modelo de atenção e cuidado dispensado às pessoas com doença falciforme. Nessa segunda visita, os técnicos brasileiros perceberam que o Senegal precisaria de capacitação para operar os equipamentos para a triagem neonatal que haviam sido doados pelo Canadá. A primeira visita de técnicos senegaleses ao Brasil ocorreu em 2008 e parece ter sido crucial para a assinatura do projeto oficial, pois nela o médico Ibrahima Diagne, responsável pelos ditames do projeto, escolheu as atividades de APS, ^3^ realizadas no Instituto de Puericultura e Pediatria Martagão Gesteira da Universidade Federal do Rio de Janeiro, como as mais adequadas ao contexto senegalês. Foi a negociação e a interação entre técnicos brasileiros e senegaleses durante essas visitas que abalou a convicção de que uma política de saúde mais restritiva seria mais apropriada ao Senegal (Brasil, 2012, p.54).

Foi a partir da primeira experiência no Senegal que os técnicos do Ministério da Saúde entenderam que seria possível fazer projetos estruturantes relativos à doença falciforme nos países de alta prevalência que não possuíam sistema de saúde gratuito e universal, como o SUS. O entusiasmo partia também da possibilidade de poder abalar a crença de que as políticas prioritárias em países subsaarianos de renda média e baixa seriam direcionadas ao controle reprodutivo de todos aqueles que apresentassem o traço ou a doença falciformes. Os brasileiros encontravam a acolhida de importantes atores atuantes na melhoria da atenção e assistência à doença falciforme em alguns países subsaarianos.

A cooperação técnica estruturante em saúde relativa à doença falciforme foi alimentada pelas relações científicas transnacionais entre cientistas e médicos especialistas na doença. O convite à participação do Brasil na conferência de Dacar, por exemplo, foi feito primeiramente ao doutor Aderson Araujo, médico da Fundação de Hematologia e Hemoterapia de Pernambuco (Hemope), que encaminhou o convite à Agência Brasileira de Cooperação por se tratar de um encontro de primeiras-damas. Antes disso, brasileiros já participavam de diversos seminários internacionais, bem como os promoviam no Brasil, como, por exemplo, os Simpósios Internacionais de Hemoglobinopatias, que começaram em 2001.

O principal ator na cooperação do Brasil com Gana, por exemplo, foi o doutor Kwaku Ohene-Frempong, médico de reconhecimento internacional, que passou a frequentar o Brasil em 2000, quando veio participar do Congresso Brasileiro de Hematologia, Hemoterapia e Terapia Celular no Rio de Janeiro. Na ocasião, conheceu a doutora Clarice Lobo, na época diretora do Instituto Estadual de Hematologia (Hemorio), onde já havia um acompanhamento estruturado às pessoas com doença falciforme, sob o suporte do Programa Estadual para Anemia Falciforme, criado em 1998 e chefiado pela doutora Joice Aragão de Jesus até sua transferência para o Ministério da Saúde, em 2004. Em setembro de 2007, quando se desenrolava a cooperação com o Senegal, Ohene-Frempong, assim como outros especialistas estrangeiros, participaram do quarto Simpósio Internacional de Hemoglobinopatias no Rio de Janeiro, cuja organização saiu do âmbito dos especialistas para a esfera do Ministério da Saúde (Lobo, 23 maio 2024).

Em abril de 2008, técnicos do Ministério da Saúde fizeram uma visita oficial a centros de pesquisa nos EUA e se encontram com o médico ganense Ohene-Frempong, que, devido à sua ampla circulação na política de seu país, deslanchou as negociações de cooperação em saúde para a doença falciforme com a República de Gana (Brasil, 2012). Outra razão para essa cooperação ter sido mais intensa foi a presença da Agência Brasileira de Cooperação em Gana, que, desde 2006, atuava na cooperação técnica em agricultura – setor que mais recebia investimentos nas Cooperações Sul-Sul (Brasil, 2011, p.40).

O primeiro acordo de cooperação deveria ajudar Gana na estruturação do Sistema Nacional de Atenção Integral à Pessoa com Doença Falciforme de Gana, “através de capacitações e treinamentos dos profissionais ganenses em programa universal de triagem neonatal” (ABC, 2010, p.69). Esse acordo teve execução rápida, pois havia muito interesse em dar os primeiros passos no projeto para a criação de um centro de hemoterapia especializado na atenção às pessoas com falciforme que fosse referência para toda a Costa Oeste da África (Brasil, 2012, p.71). O problema da qualidade do sangue era crucial nas tratativas de cooperação, tendo em vista sua importância para o tratamento na doença falciforme.

Entre abril e dezembro de 2009, ocorreu a formatação desses dois projetos de cooperação com Gana. A execução do primeiro foi rápida e se desdobrou no Brasil com o treinamento de técnicas(os) ganenses na triagem neonatal pelo Núcleo de Ações e Pesquisa em Apoio Diagnóstico da Faculdade de Medicina da Universidade Federal de Minas Gerais e pela Fundação Centro de Hematologia e Hemoterapia do Estado de Minas Gerais (Hemominas) e de enfermeiras(os) no modelo de atenção integral à saúde ofertado pelo SUS (Brasil, 2012, p.70-73). A triagem neonatal começou em 1995 em Gana, uma das nações pioneiras no continente africano a executar esse serviço. Em dez anos, foram testados mais de duzentos mil recém-nascidos, tendo quase 2% desse total apresentado doença falciforme (Ohene-Frempong et al., 2008). Não havia, no entanto, educação continuada entre os profissionais de saúde, o que motivou a cooperação brasileira para a capacitação de trabalhadores.

Existia um grande entusiasmo com a disponibilidade de recursos mínimos e críticos indispensáveis às crianças com doença falciforme no Brasil – triagem neonatal, penicilina de rotina e vacinas – que se expressava nas conversas entre os brasileiros e os médicos-cientistas ganenses Kwaku Ohene-Frempong e Isaac Odame, radicados nos EUA e no Canadá, respectivamente (Jesus, Cortez, 14 maio 2024; Araujo, 14 jun. 2024). O doutor Aderson Araujo relatou que havia esse entusiasmo em entender como funcionava o SUS, mencionando que em um congresso internacional foi chamado pelo próprio Isaac Odame para contar a outros especialistas a experiência brasileira no tratamento de pessoas com doença falciforme (Araujo, 14 jun. 2024). Odame, por sua vez, externava em publicações o exemplo do Brasil como um país de média renda que provia o mínimo para a sobrevivência das crianças com menos de 5 anos e oferecia gratuitamente hidroxiureia a uma considerável população de adultos (Aygun, Odame, 2012).

O diálogo com os atores senegaleses e ganenses e as tratativas dos acordos de cooperação já ocorriam intensamente em janeiro de 2009, quando uma comitiva de especialistas brasileiros participou da reunião Advancing Sickle Cell Disease Patient Care Through Global Research na capital da República do Benim, Cotonou. Participantes de outros 24 países aderiram à reunião que pretendia estabelecer parcerias e cooperações de longo prazo entre países de alta, média e baixa rendas para “avançar nas pesquisas e incrementar globalmente a assistência médica” (Odame, 2010, p.571). Nota-se que não há menção a triagem populacional, testes pré-maritais, abortos seletivos etc. entre os seis grupos de trabalho que trataram dos seguintes pontos: triagem neonatal, educação, aconselhamento e cuidado; doenças infecciosas na doença falciforme; terapia com hidroxiureia na África e outras regiões em desenvolvimento; abordagem global para os fatores genéticos envolvidos na diversidade fenotípica; criação de competências por meio de uma rede global de trabalho sobre a doença falciforme para se ter um plano de ação; e discussões sobre a experiência em projetos colaborativos para estabelecer centros de doença falciforme em países de baixa renda (Odame, 2010). Embora não possa afirmar que a prevenção de nascimentos não esteve em debate no congresso, percebo que o assunto não recebeu foco prioritário nessa reunião como vinha ocorrendo em eventos como o terceiro Congresso da Organização Internacional de Combate à Doença Falciforme, em Dacar, em 2006, e em publicações internacionais como artigos científicos e documentos da OMS.

No evento de Cotonou, o grupo brasileiro sugeriu que a doença falciforme fosse usada como motivo para estruturar um sistema de saúde nos moldes do SUS em países da África com alta prevalência da doença. ^4^ Era uma estratégia para que houvesse serviços de saúde nos níveis primário, secundário e terciário (Brasil, 2012, p.82). Aproveitando a recente assinatura de um acordo de cooperação em saúde, o grupo de brasileiros foi chamado para uma reunião com o ministro da saúde beninense e o embaixador brasileiro a fim de iniciar um projeto para a doença falciforme. Em dois meses, Benim e Brasil assinaram o Projeto-Piloto em Doença Falciforme, pelo qual técnicos beninenses foram treinados no Brasil – na triagem neonatal no Núcleo de Ações e Pesquisa em Apoio Diagnóstico da Universidade Federal de Minas Gerais e no Hemominas (p.82-83) – e técnicos brasileiros mediaram a capacitação nos equipamentos de triagem neonatal cedidos ao Benim. A melhoria na área da hemoterapia também era uma necessidade precípua, em razão do uso do sangue como tratamento, e fez parte dessa cooperação (Januário, 28 maio 2024).

Assim como em Gana, no Benim já havia experiência em triagem neonatal desde os anos 1990 (Rahimy et al., 2003). O doutor Mohamed Cherif Rahimy, que esteve à frente dessa cooperação técnica com o Brasil, realizou, entre 1993 e 1999, estudo sobre o acompanhamento de crianças em um programa integral de atenção à saúde, revelando a importância desse programa para a diminuição da morbidade e da mortalidade. Outras conclusões importantes foram: a percepção da necessidade de se ter equipes treinadas e comprometidas; e a constatação de que o programa não demandara significativo aporte de recursos e, portanto, poderia ser implementado em localidades com poucos recursos (Rahimy et al., 2003, p.838). Apesar de a ausência de estruturas básicas de saúde ser um dos grandes obstáculos para implementar programas como esse nos países da África subsaariana, a falta de educação médica era um dos óbices mais significativos, pois impedia os diagnósticos e os consequentes acompanhamentos vitais à sobrevivência das crianças abaixo dos 5 anos.

É preciso destacar a importância de Isaac Odame e Kwaku Ohene-Frempong no cenário internacional e as suas atuações como divulgadores da experiência brasileira. Em 2010, ambos participaram da criação da Global Sickle Cell Disease Network, ^5^ cuja primeira conferência ocorreu em 2011, em Acra, Gana, e a segunda, em 2014, no Rio de Janeiro, tendo sido organizada pelo Hemorio e pela Coordenadoria-geral de Sangue e Hemoderivados do Ministério da Saúde do Brasil. Segundo Odame, Kulkarni e Ohenefrempong (2011, p.417), essa organização internacional foi criada para que as pessoas com doença falciforme fossem representadas de maneira mais robusta, assim como se dava no caso da Federação Internacional de Talassemia. A ideia foi aventada no evento promovido por essa federação e pela OMS em novembro de 2007, Management of Haemoglobin Disorders: Report of a Joint WHO-TIF Meeting.

Voltemos à análise da primeira seção, sobre a comparação entre a talassemia e a doença falciforme. A existência de uma organização internacional para defender as pessoas com doença falciforme era necessária e essencial para seguir com os processos de conscientização e educação sobre a doença em sentido amplo. A experiência mobilizadora da Federação Internacional de Talassemia pode continuar a ser um exemplo, mas, dadas as enormes diferenças entre as doenças, ainda que sejam ambas hemoglobinopatias, é preciso questionar se as políticas prioritárias precisam ser iguais.

A cooperação técnica estruturante em saúde para a doença falciforme que o Brasil construiu junto ao Benim, a Gana e ao Senegal mostrava que era possível, mesmo em locais de recursos limitados, prover saúde e acompanhamento nos primeiros anos de vida das crianças nascidas com doença falciforme. As principais ações das cooperações se concentraram na capacitação de profissionais para a triagem neonatal, que é a condição básica para que qualquer programa de tratamento e melhoria da qualidade de vida possa ser implementado. Segundo os atores brasileiros que participaram dessas cooperações, a fama dos programas brasileiros de vacinação e aleitamento materno facilitou o interesse pelas parcerias técnicas estruturantes em saúde para a doença falciforme (Jesus, Cortez, 14 maio 2024; Januário, 28 maio 2024).

## Considerações finais

A priorização de uma atenção integral à saúde para as pessoas com doença falciforme, em oposição ao controle reprodutivo, revela não apenas um posicionamento ético em relação às pessoas com traço e doença falciformes, mas também as oportunidades políticas daquele momento no certame das Cooperações Sul-Sul, em cujo cenário a política externa do Brasil de aproximação aos países em desenvolvimento buscava fortalecer o papel do país internacionalmente. O enquadramento de uma política global de prevenção da doença falciforme no século XXI ocorreu em paralelo às cooperações técnicas em saúde que deram visibilidade à política nacional brasileira para as pessoas com essa doença, que só funcionava devido à existência do SUS. A intenção das cooperações brasileiras em saúde era difundir seu modelo de APS (Almeida et al., 2010) e, no caso da doença falciforme, realizar uma oposição à política de prevenção de nascimentos. O investimento conjunto do Ministério das Relações Exteriores e do Ministério da Saúde nessa cooperação da doença falciforme não foi tímido e ficou atrás apenas do atribuído aos projetos relativos à aids (Tagliari, 2014). ^6^

O papel do Brasil na demonstração de que era possível cuidar de pessoas com doença falciforme em um país de média renda foi importante para sensibilizar nações como Benim, Gana e Senegal, que, embora já tivessem experiências nesse sentido, não tinham conhecimento do acompanhamento dessas pessoas nas estruturas de saúde brasileiras. A questão dos recursos necessários ao tratamento de pessoas com doença falciforme sempre é apresentada de maneira simplificada, como se todos os contextos fossem iguais. A atuação do Brasil mostrou ser possível oferecer acompanhamento adequado quando já havia um sistema de saúde estruturado – ainda que precário – e que esse acompanhamento diminuía os custos com a doença devido à contenção das intercorrências. Conforme explica a doutora Joice Aragão de Jesus, os custos de tratamento das pessoas com doença falciforme estão embutidos no SUS, ou seja, são repartidos por outros milhares de usuários com diferentes doenças e agravos à saúde, pois não existe tratamento, exame ou procedimento único e específico para a doença falciforme (Jesus, Cortez, 14 maio 2024).

O esforço de cooperação técnica em saúde do Brasil trazia em si muitos processos históricos em andamento nas arenas da saúde global (consolidação da primazia das métricas, da securitização e da accountability), na política nacional de saúde (consolidação de um sistema de saúde gratuito e universal) e na longa história da interpretação médico-científica da doença falciforme (mudança na definição de doença hereditária do sangue para defeito de nascença evitável). Numa esfera da arena internacional, na qual a OMS e outras organizações internacionais atuam em parceria com Estados nacionais, promoviam-se as políticas de controle das populações com traço e doença falciformes, ainda que reconhecendo a importância do acesso à saúde para as pessoas com doença falciforme. Em outra esfera, vimos a disseminação do modelo brasileiro de saúde ofertado às pessoas com doença falciforme como uma iniciativa de divulgação e intercâmbio dos valores universalistas e igualitários da reforma sanitária brasileira que estão legalmente presentes no SUS.

## Data Availability

Não estão em repositório.

## Notas

1 Realizei quatro entrevistas sob o respaldo do Certificado de Apresentação de Apreciação Ética: 73202323.6.0000.5241, obtido em 16 de novembro de 2023 do Comitê de Ética em Pesquisa da Escola Politécnica de Saúde Joaquim Venâncio/Fiocruz.

2 As demais visitas de prospecção aconteceram na Guiné-Bissau, em 2007, não resultando em cooperação devido à instabilidade político-econômica da época; na Jamaica, em julho de 2010, que também não resultou em cooperação em razão da inadequação da demanda. Angola recebeu cooperação do Brasil na área da doença falciforme (Brasil, 2012, p.60, 65, 86).

3 APS aqui compreende as atividades de prevenção das intercorrências no atendimento iniciado com os recém-nascidos diagnosticados com a doença e a promoção do autocuidado nos adultos.

4 O grupo brasileiro era formado por Anna Bárbara Proeitti (Hemominas), Aderson Araujo (Hemope), Clarice Lobo (Hemorio), Guilherme Genovez (CGSH), Joice Aragão de Jesus (PNAIPDF), José Nélio Januário (Nupad/UFMG), Marilda Gonçalves (Fiocruz/Bahia), Paulo Ivo Cortez (UFRJ).

5 Disponível em: https://www.globalsicklecelldisease.org/about-sickle-cell-disease . Acesso em: 2 out. 2024.

6 Embora pareça que o investimento na cooperação técnica em doença falciforme estivesse, assim como o aportado para a aids, centrado em uma doença específica (Tagliari, 2014), na verdade, ele se diversificou em ações de capacitação para a triagem neonatal e a melhoria da oferta de sangue, ou seja, para ações de serviços básicos de saúde (Brasil, 2012; Januário, 28 maio 2024).
